# Exploring the telehealth readiness and its related factors among palliative care specialist nurses: a cross-sectional study in China

**DOI:** 10.1186/s12904-023-01209-1

**Published:** 2023-06-28

**Authors:** Junchen Guo, Yunyun Dai, Youwen Gong, Xianghua Xu, Yongyi Chen

**Affiliations:** 1 Department of Palliative care, Hunan Cancer Hospital, No.283, Tongzipo Road, Yuelu District, Changsha, 410006 Hunan China; 2 School of Nursing, University of South China, No.28, Changsheng West Road, Hengyang, 421001 Hunan China; 3 Health Services Research Institute, University of Wollongong, Northfields Avenue, Wollongong, NSW 2522 Australia; 4 Department of Nursing, The First People’s Hospital of Changde City, Changde, China

**Keywords:** Telehealth readiness, Palliative care, Specialist nurses

## Abstract

**Backgrounds:**

The majority of Chinese people who are nearing the end of their lives prefer to receive home-based palliative care. Telehealth, as a new service model, has the potential to meet the increasing demand for this service, especially in remote areas with limited resources. However, nurse-led telehealth-based palliative care services are still in the pilot implementation phase. Assessing the telehealth readiness among palliative care specialist nurses and identifying associated factors is crucial to facilitate the successful implementation of telehealth services. Therefore, this study aimed to examine TH readiness and its related factors among Chinese palliative care specialist nurses.

**Methods:**

Four hundred nine Chinese palliative care specialist nurses from 28 provinces or municipalities participated in this study between July and August 2022. The Chinese version of Telehealth Readiness Assessment Tools (TRAT-C), and Innovative Self-Efficacy Scale (ISES-C) were used to assess the degree of TH readiness and the levels of innovative self-efficacy.

**Results:**

The total score of the TRAT-C was 65.31 ± 9.09, and the total score of ISES was 29.27 ± 5.78. The statistically significant factors that influenced telehealth readiness were the experience of using telehealth platforms or services, the willingness to provide telehealth to patients, and the level of nurses’ innovative self-efficacy. The innovative self-efficacy is positively correlated to telehealth readiness (*r* = 0.482, *P* < 0.01). These related factors could explain 27.3% of the difference in telehealth readiness.

**Conclusion:**

The telehealth readiness of Chinese palliative care specialist nurses are at a moderate level. Measures such as providing incentives to promote nurses’ innovation self-efficacy by nurse managers, and establishing a comprehensive telehealth training system for palliative care specialist nurses should be taken to facilitate the implementation of telehealth services in the field of palliative care.

**Supplementary Information:**

The online version contains supplementary material available at 10.1186/s12904-023-01209-1.

## Background

Palliative care aims to improve the quality of life for patients and their families facing life-threatening illnesses by preventing and reducing suffering through early detection and treatment of pain and other issues [1]. According to the World Health Organization (WHO), over 56.8 million people require palliative care each year, including 25.7 million in the last year of their life [2]. However, only 14% of patients globally have access to palliative care, and this number is expected to almost double by 2060 due to the increasing number of the ageing population and the incidence of the non-communicable disease [2].

In China, there are 10.4 million of the population in need of palliative and end-of-life care, accounting for 17% of the total people globally, according to the Serious Health-Related Suffering (SHS) Database, which is produced by the Lancet Commission on Global Access to Palliative Care and Pain Relief (GAPCPR) [3]. The majority of those nearing the end of their lives in China prefer to receive palliative care at home and have expressed their desire for nurses to provide continuity of high-quality palliative care services [4, 5]. However, there are challenges for nurses providing home-based palliative care due to the limited number of palliative care specialist nurses and the increased workload of travelling to rural and remote areas [6, 7]. There is a need for a system to reduce workload while ensuring that patients receive satisfactory healthcare irrespective of their geographical locations.

With the rapid integration of global information and communication technology into the field of the healthcare system, a healthcare service model - telehealth (TH), defined as the use of information technology by health professionals in remote contexts to support and promote clinical healthcare, patient and professional health-related education, and health administration, has vastly expanded the scope of healthcare services [8]. TH provides an opportunity for efficient communication, continuity of care, and meets the growing demands of care services across remote areas with limited resources [9]. Previous studies reported that TH could potentially reduce unnecessary hospital admissions and increase end-of-life patients’ quality of life by improving their independence, self-management, and access to high-quality of home-based palliative care services [10–12]. In 2022, the National Health Commission of the People’s Republic of China issued the National Nursing Career Plan 2021 to 2025, which clearly states that nursing services should be innovated with the full use of TH to provide convenient and efficient care for palliative care patients [13]. Nurse-led TH-based palliative care services have increased with the advancement of relevant policies [14, 15].

The readiness of healthcare professionals plays a vital role in the successful application of the TH services [16]. However, nurse-led telehealth-based palliative care services are still in the pilot implementation phase in China. It is necessary to comprehensively evaluate and discuss the TH readiness among palliative care specialist nurses and develop training programs to ensure the successful application of TH. Innovative self-efficacy, defined as the individual’s belief in their ability to innovate and adopt new technology [17], was identified as one of the most critical factors related to the TH readiness of healthcare professionals [18]. Healthcare professionals with a high level of innovative self-efficacy were more willing to participate in and successfully adopt the TH services, as the high level of innovative self-efficacy could unleash their creative thinking, stimulate active exploration of new things, such as new innovative methods and technologies of care, and will continue to turn these innovative ideas into reality through practice [18]. However, the current situation of TH readiness and innovation self-efficacy among Chinese palliative care nurses has yet to be investigated, which hinders the ability to develop tailored interventions to improve their TH readiness. Therefore, this study aims to (1) Examine the level of TH readiness among Chinese palliative care specialist nurses; (2) Assess the level of innovative self-efficacy among Chinese palliative care specialist nurses; and (3) Explore the relationship between innovative self-efficacy and TH readiness among Chinese palliative care specialist nurses.

## Methods

### Study design

This is a descriptive cross-sectional study that was performed in China. The research conformed to the Strengthening the Reporting of Observational Studies in Epidemiology (STROBE) Statement: guidelines for reporting observational studies.

### Participants and sample size

Using convenience sampling method, we recruited participants in the Chinese Nursing Association (CNA) Palliative Care Nurse Specialist Training Base between July and August 2022. The inclusion criteria for participants included: (1) registered nurse; and (2) participated in the Palliative Care National Nurse Specialist Training Course, which organized by the Palliative Care Committee of the CNA, and successfully obtained the palliative care specialist nurse qualification certificate. The exclusion criteria were as the following: (1) refused to participate in this study, and (2) cognitive impairment and inability to answer relevant questions. The sample size calculation was based on the formula N ≥ 50 + 8 m (m is the number of independent variables) to test for multiple regression with the desired power = 0.8, type I error = 0.05 [19]. There were 16 variables in our study, thus, the sample size was: *N* = 50 + 8*16 = 178, and assuming 20% of questionnaires were invalid, at least 214 participants were required in this study.

### Measures

#### Demographic information questionnaire

We designed a questionnaire to collect the participant’s demographic information that included gender, age, marital status, educational background, hospital level, professional title, monthly income (RMB), employment category, and years of experience in palliative care. Additionally, we developed a simple questionnaire to investigate the participant’s willingness to participate in TH by asking the following questions: “Have you learned about telehealth services?“ “Have you used telehealth platforms or services?“, and “Are you willing to provide telehealth services to patients?“ (Appendix I).

#### Telehealth readiness

Jennett et al. developed the Telehealth Readiness Assessment Tool (TRAT), a self-reporting scale to measure nurses’ TH readiness [20]. The scale contains 17 items consisting of these three domains: core readiness, engagement readiness, and structural readiness. All items are scored on a 5-point Likert scale from “very disagree” to “very agree”. The score ranges from 17 to 85. We divided TH readiness into these three levels: high level (> 80), which indicates that practitioners can use TH well; moderate level (61–80), which indicates that certain factors may adversely affect their ability to implement TH; and low level (< 61), which indicates that practitioners have many barriers to successfully implementing TH. In this study, we used the Chinese version of the TRAT (Additional file 2), which was culturally adapted and validated by the Chinese researcher Liu and her colleagues [21]. The Chinese version of the TRAT had a good reliability and validity, with the content validity index (S-CVI) was 0.93, and the item-level content validity index (I-CVI) was 0.88 to 1.00, the Cronbach’s alpha coefficient was 0.861 [21]. In our study, Cronbach’s alpha was 0.853.

#### Innovative self-efficacy

The Innovative Self-Efficacy Scale (ISES) is an 8-item scale developed by Carmeli et al. [22], which includes the following statements: “I can accomplish the majority of my goals through innovative ways of working.“ “Faced with difficult tasks, I’m pretty sure I’ll be able to do it creatively.“ “Overall, I feel that I can innovatively make significant achievements.“ “Most of the time, I can turn an innovative idea into a reality.“ “I can deal with all kinds of challenges creatively.“ “I am confident in my creative ability to accomplish various tasks.“ “Compared to others, I am very creative in my work.“, and “Even when things are difficult, I still do it creatively.“ All items are scored on a 5-point Likert scale from “very disagree” to “very agree”. And the total score ranges from 8 to 40, with higher scores indicating higher levels of innovative self-efficacy. This study employed the Chinese version of the ISES (Additional file 3), which was cross-culturally adapted and validated by Gu et al., to assess the level of innovative self-efficacy among Chinese palliative care specialist nurses. It had good reliability, with Cronbach’s alpha of 0.886 [23] . In our study, Cronbach’s α was 0.967.

#### Data collection

The participant consent form, the Chinese version of the TRAT, the ISES, together with the demographic information questionnaire were imported into Questionnaire Star (a Chinese professional online data collection platform) to produce an online survey link, which was then distributed to the CNA Palliative Care Nurse Specialist Training WeChat Group. On the first page of this survey, participants were directed to read the contents of the participant informed consent form, which included detailed information about the purposes of this study, the time that may be spent, the possible risks and benefits associated with participation, as well as avenues for raising complaints about this study. Following the review of the informed consent form, participants were presented with a single question, “Would you like to participate in this study?“, with the answer options “Yes” or “No”. Selection of “Yes” indicated that nurses were willing to participate in this study and would then be directed to the second page to complete the survey. The survey link was available for 13 days (from 31 to 2022 to 12 August 2022). Each question was set to the mandatory answer, and each IP address and account could only complete the survey one time to avoid the duplication. To ensure high-quality data, two researchers who were very familiar with the research topic completed the questionnaire before the formal survey. The minimum time spent on completing the survey by them was 120 s. The link to the survey was clicked 415 times, and responses completed in a very short time were excluded (< 120 s). Finally, we collected 409 valid questionnaires (response rate: 98.55%).

### Statistical analysis

The IBM SPSS Statistics (Version 25.0. IBM Corp) was used to perform the statistical data analysis in our study. The categorical data were described by calculating frequencies and percentages, the continuous data were described as the mean and standard deviation (SD). The Kolmogorov- Smirnov test was used to examine the normality of the continuous data distribution. One-way ANOVAs and *t*-tests were used to determine factors associated with TH readiness among nurses initially, and Spearman correlation analysis was conducted to detect a relationship between the levels of nurses’innovative self-efficacy and their TH readiness due to the score of the innovative self-efficacy did not obey the normal distribution in our study. We used multivariate linear regression to explore the associated factors with the TH readiness. We conducted all statistical tests as two-tailed and considered a *P*-value of less than 0.05 statistically significant.

### Ethical considerations

The study was approved by the Human Research Ethics Committee of Hunan Cancer Hospital (No 2022-75). This study was conducted according to the principles of the Declaration of Helsinki and followed relevant guidelines and regulations. Informed consent was obtained from all participants before they participated in the online survey.

## Results

### Demographic information

A total of 409 palliative care specialist nurses from 28 provinces or municipalities across China participated in this study. Females made up 98.5% (*n* = 403) of the group, 15 nurses were from primary hospitals, 38 nurses were from secondary hospitals, and 356 were from tertiary hospitals. The demographics of participants were presented in Table 1.

**Table 1:** Demographic Information of Palliative Care Specialist Nurses (*N* = 409)

Variables	Categories	n (%)	TH Readiness	*t*/*F*	*P*
			M(SD)		
Age	20-30year	89(21.8)	65.66(9.19)	0.092	0.912
	31-40year	263(64.3)	65.24(9.08)		
	≥ 41year	57(13.9)	65.09(9.12)		
Marital status	Unmarried	61(14.9)	65.61(9.07)	0.024	0.877
	Married	348(85.1)	65.26(9.11)		
Educational background	Junior college and below	24(5.9)	64.58(9.12)	1.341	0.263
	Bachelor’s degree	366(89.5)	65.19(9.09)		
	Master’s degree and above	19(4.6)	68.58(8.83)		
Hospital level	Tertiary hospitals	356(87.0)	65.57(9.23)	1.533	0.217
	Secondary hospitals	38(9.3)	64.24(8.08)		
	Primary hospitals	15(3.7)	61.80(7.46)		
Professional title	Nurse	12(2.9)	63.50(9.14)	1.302	0.273
	Senior nurse	159(38.9)	65.89(9.45)		
	Supervisor nurse	206(50.4)	64.64(8.89)		
	Deputy chief nurse and above	32(7.8)	67.38(8.35)		
Monthly income (RMB)	≤ 5,000	80 (19.5)	64.46(10.34)	0.482	0.618
	5,001–10,000	242(59.2)	65.61(8.86)		
	≥ 10,000	87(21.3)	65.24(8.54)		
Employment category	Contract employee	241(58.9)	64.94(9.08)	0.769	0.464
	Personnel agency employee	38(9.3)	66.82(8.98)		
	Formal employee	130(31.8)	65.55(9.16)		
Working experience	≤ 1year	170(41.6)	64.21(9.22)	2.084	0.102
	2-5year	143(35.0)	65.47(8.63)		
	5-10year	58(14.1)	66.59(9.12)		
	≥ 10year	38(9.3)	67.66(9.75)		
Experience of learning TH	Yes	177(43.3)	67.81(9.34)	4.998	< 0.001**
	No	232(56.7)	63.40(8.43)		
Experience of using TH platforms or services	Yes	194(47.4)	68.08(9.07)	6.107	< 0.001**
	No	215(52.6)	62.81(8.38)		
TH service willingness	Yes	381(93.2)	65.78(8.90)	3.960	< 0.001**
	No	28(6.8)	58.86(9.38)		

Independent sample *t*-test and ANOVA analysis were used to compare the mean score of TH readiness. **indicates *P *< 0.001

### The telehealth readiness and the innovative self-efficacy of chinese palliative care specialist nurses

The total score of TRAT-C was 65.31 ± 9.09 (range from 20 to 85), which indicated that Chinese palliative care specialist nurses had a moderate level of readiness to provide the telehealth services to patients. The mean scores of each domain of the TRAT-C were showed in Table 2. The total score of the ISES-C and the average score for each item were presented in Table 2.

**Table 2:** Mean Scores of TRAT-C and ISES-C (*N* = 409)

Variable	Numbers of items	Score	Range	Item average score
		M(SD)		M(SD)
TRAT-C total	17	65.31(9.09)	20–85	3.84(0.53)
Core readiness	4	14.74(2.46)	6–20	3.69(0.62)
Engagement readiness	7	26.94(4.69)	8–35	3.85(0.67)
Structural readiness	6	23.63(3.94)	6–30	3.94(0.66)
ISES-C total	8	29.27(5.78)	8–40	3.66(0.72)

### Relationship between innovative self-efficacy and TH Readiness

The Spearman correlation indicates that innovative self-efficacy is positively correlated to TH readiness (*r* = 0.482, *P* < 0.01) (Table 3).

**Table 3:** Relationship Between Innovative Self-efficacy and TH Readiness (*N* = 409)

Variable	TRAT	Core readiness	Engagement readiness	Structural readiness
ISES	0.482**	0.251**	0.488**	0.385**

**indicates *P* < 0.01

### Factors related to nurses’ TH readiness

Table 1 shows the results of the univariate analysis. Nurses who had learned about TH (*t* = 4.998, *P* < 0.001), used TH platforms or services (*t* = 6.107, *P* < 0.001), and expressed willingness to provide TH services to patients (*t* = 3.960, *P* < 0.001) had a higher score in TH readiness. We did not find significant differences in TRAT-C scores by age, marital status, educational background, hospital level, professional title, monthly income, employment category or working experience.

Taking TH readiness as the dependent variable, the statistically significant variables that presented both in Tables 1 and 3 as the independent variables, multiple linear regression analysis was used to examine the factors associated with nurses’ TH readiness. The specific assignment for independent variables were shown in Table 4. The linear regression equations fit the data well (*F* = 39.244, *P* < 0.001), which explained 27.3% of the variances. The experience of using TH platforms or services (*β* = 0.162, *P* = 0.002), willingness to provide TH to patients (*β* = 0.136, *P* = 0.002), and the level of innovative self-efficacy (*β* = 0.417, *P* < 0.001) were the factors that influenced TH readiness (Table 5).

**Table 4:** Assignment of independent variables

Variable	Assignment method
Experience of learning TH	0 = No, 1 = Yes
Experience of using TH platforms or services	0 = No, 1 = Yes
TH service willingness	0 = No, 1 = Yes
Innovative self-efficacy	Measure value

**Table 5:** Multiple Linear Regression Analysis of TH Readiness

Variable	*B*	*SE*	*β﻿*	*t﻿*	*P*
Constant	39.648	2.393	-	16.566	< 0.001**
Innovative self-efficacy	0.656	0.068	0.417	9.719	< 0.001**
Experience of using TH platforms or services	2.944	0.948	0.162	3.106	0.002*
TH service willingness	4.901	1.545	0.136	3.173	0.002*

*R* = 0.529, *R* ^2^ = 0.280, Adjusted *R* ^2^ = 0.273, *F* = 39.244, *P* < 0.001

*indicates *P* < 0.05, **indicates *P* < 0.001

## Discussion

To the best of our knowledge, this is the first study to explore the TH readiness and its related factors among Chinese palliative care specialist nurses. The total TRAT-C score of palliative care specialist nurses was 65.31 ± 9.09 in our study, which indicated that they had a moderate level of TH readiness and they encountered certain factors that could adversely affect their ability to implement TH. Palliative care specialist nurses who had used TH platforms or services, expressed willingness to provide TH services to patients and had a higher level of innovative self-efficacy exhibited a higher degree of readiness to implement TH.

Recently, nurse-led TH-based palliative care services have increased with the advancement of Chinese government policies on the uptake of TH to provide convenient and efficient care for palliative care patients [13]. However, the finding of this study suggested that the level of TH readiness among Chinese palliative care specialist nurses are still at a moderate level, indicating that there are still barriers hindering the adoption or the implementation of TH in the field of palliative care. Prendergast et al. reported that lack of TH training was one of the obstacles for nurses to implement TH [24]. Currently, there are no standardized TH training courses available for specialist palliative care nurses in China. Nurse may lack access to the necessary knowledge about TH concepts and specific clinical practice models, which in turn impacts their perception of the usefulness of TH and their readiness to provide TH services. In 2019, List and his team integrated TH into a traditional Nurse Practitioner Training Program, and developed learning outcomes and a one-hour didactic TH presentation intervention, which significantly improved the confidence levels in TH knowledge and the readiness [25]. Therefore, It is crucial that future research focuses on developing a comprehensive Chinese TH training curriculum system to strengthen nurses’ perception of TH and enhance their level of readiness for providing TH services. A similar study was conducted by Muigg et al. in 2017, which examined the TH readiness was 58.2 (95% CI 53.9–62.5) among 41 Australian medical practitioners in diabetes care, slightly lower than that of Chinese palliative care nurses [26]. In addition to different sample sizes, the difference in results may be due to the fact that our study was conducted during the prevalence of Coronavirus disease 2019 (COVID-19). Face-to-face care services were extremely limited in China between 2020 and 2022 due to the COVID-19 pandemic [27]. During this period, the Chinese government provided strong support and promotion for TH services [28, 29]. TH services have proven to meet patients’ needs for medical care and improve the utilization of human resources for healthcare during the epidemic, further accelerating the development and implementation of TH services in China [30, 31]. It may explain why our participants’ had slightly higher scores on TH readiness than in other studies.

Of the factors studied, we found that nurses who had previously used TH platforms or services had higher scores on TRAT-C than those who had not. This may be due to the fact that nurses with previous experience of using TH platforms or services have a deep and clear understanding of the benefits of TH and have accumulated valuable experience in engagement and successful implementation. To some extent, this could enhance the readiness and capability of providing TH services to palliative care patients. In addition, we found that nurses who were willing to provide TH for patients had higher scores on TRAT-C than the counterparters, which was supported by a study conducted by Tian et al. [27]. In our study, 93.2% of palliative care specialist nurses were willing to provide TH for patients, demonstrated that the majority of them had a positive attitude towards TH. China is currently struggling with a challenge where the limited number of palliative care specialist nurses cannot meet the increased demand for home-based palliative care, as most patients prefer to die at home [6]. TH-based palliative care services may be considered as a potential solution to alleviate the current shortage of palliative care specialist nurses. Furthermore, compared to traditional face-to-face home care, the implementation of TH-based palliative care services have been shown to save healthcare costs, enhance nurse’work efficiency, and improve job satisfaction [32, 33]. As the nurses gain more experience and benefits with TH service, they may gradually recognize its usefulness and availability, which can increase their willingness to incorporate TH in their clinical practice.

In our study, palliative care specialist nurses had a moderate to high level of innovative self-efficacy. The level of innovative self-efficacy of this study is slightly higher than that of non-specialist clinical nurses [34]. The reason may be that the responsibility of specialist nurses to adopt the nursing technology innovation, conduct research, and resolve various challenging problems related to nursing service, they are more likely to generate more creative thinking than the non-specialist clinical nurses [34]. Additionally, our findings suggested that palliative care specialist nurses who had a higher level of innovative self-efficacy with a higher degree of the TH readiness. Nurses with a strong sense of innovative self-efficacy tend to be more open to new methods and technologies, and they are more likely to embrace change and view new technology as an opportunity rather than a challenge. Moreover, they are more inclined to proactively seek out information and resources to support their learning and development, leading to more innovative behaviors. They are willing to overcome the difficulties encountered in the innovation process and constantly explore new methods to solve the problems that arise in their clinical practice [18, 35]. Innovation is vital in ensuring organizational success and sustainability in an increasingly competitive work environment [36]. McHugh et al. pointed out that the innovative capability of nurses can improve the quality of patient care [37]. Therefore, nursing managers can develop an innovative culture and provide incentives to improve the level of TH readiness of palliative care specialist nurses by promoting the innovation self-efficacy level.

### Limitations

There were limitations to this study. First, the TART used in our study to assess the TH readiness is a self-report scale, which could involve recall and social desirability bias. Additionally, we used a cross-sectional survey that could not track the changes in the TH readiness of nurses over time. Future study will consider to conduct a longitudinal study to explore the changes in TH readiness among nurses over time.

## Conclusions

With the increased demanding of home-based palliative care and the limited number of palliative care specialist nurses in China, TH is considered one of the most important alternative approaches to balancing conflicts between demand for and the supply of home-based palliative care. However, the TH readiness among Chinese palliative care specialist nurses needs to be improved. The experience of using TH platforms or services, the willingness to provide TH services, and the innovative self-efficacy of nurses were closely related to the TH readiness. Measures such as providing incentives to promote nurses’ innovation self-efficacy by nurse managers, and establishing a comprehensive telehealth training system for palliative care specialist nurses should be taken to facilitate the implementation of telehealth services in the field of palliative care.

## Supplementary Information

**Additional file 1.**

**Additional file 2.**

**Additional file 3.**

## Data Availability

The data used during this study are available from the corresponding authors on reasonable request.
