# Supplementary material for: Exploring the telehealth readiness and its related factors among palliative care specialist nurses: a cross-sectional study in China
Source: BMC Palliat Care. 2023 Jun 28;22:82. doi: 10.1186/s12904-023-01209-1 (PMC10303327; doi:10.1186/s12904-023-01209-1)
Supplement: Supplementary file 1 — Additional file 1. [file 12904_2023_1209_MOESM1_ESM.docx]

**Appendix I Demographic Information Questionnaire**

1.What is your gender？

□Male □Female

2.What is your age?

□20-30year □31-40year □41-50year □≥50year

3.What is your marital status?

□Unmarried □Married □Divorced □Widowed

4.What is your educational background?

□Junior college and below □Bachelor’s degree □Master’s degree

□Ph.D. degree and above

5.What is your hospital level?

□Tertiary hospitals □Secondary hospitals □Primary hospitals

6.What is your professional title?

□Nurse □Senior nurse □Supervisor nurse □Deputy chief nurse and above

7.What's your monthly income(RMB)?

□≤3,000 □3,000-5,000 □5,000-10,000 □≥10,000

8.What's your employment category?

□Contract employee □Personnel agency employee □Formal employee

□Temporary employee

9.How long have you worked in palliative care?

□≤1year □2-5year □5-10year □≥10year

10.Have you learned about telehealth services?

□Yes □NO

11.Have you used to telehealth platforms or services?

□Yes □NO

1. Are you willing to provide telehealth services to patients?

□Yes □NO
