# Supplementary material for: Exploring the telehealth readiness and its related factors among palliative care specialist nurses: a cross-sectional study in China
Source: BMC Palliat Care. 2023 Jun 28;22:82. doi: 10.1186/s12904-023-01209-1 (PMC10303327; doi:10.1186/s12904-023-01209-1)
Supplement: Supplementary file 2 — Additional file 2. [file 12904_2023_1209_MOESM2_ESM.docx]

**Appendix** **II**

**Chinese version of the Telehealth Readiness Assessment Tool (TRAT)**

1.I am not satisfied with the way care is provided.

□Very disagree

□Somewhat disagree

□Uncertain

□Somewhat agree

□Very agree

2.I have experienced firsthand the negative impact of health resource shortages.

□Very disagree

□Somewhat disagree

□Uncertain

□Somewhat agree

□Very agree

3.There is an urgent need for me to address the care of my patients through telehealth.

□Very disagree

□Somewhat disagree

□Uncertain

□Somewhat agree

□Very agree

4.I am an innovator and advocate of telehealth services.

□Very disagree

□Somewhat disagree

□Uncertain

□Somewhat agree

□Very agree

5.I am interested in exploring the impact of telehealth services in improving healthcare patterns.

□Very disagree

□Somewhat disagree

□Uncertain

□Somewhat agree

□Very agree

6.I respect every member of the telehealth service team.

□Very disagree

□Somewhat disagree

□Uncertain

□Somewhat agree

□Very agree

7.I need to interact with other telehealth providers.

□Very disagree

□Somewhat disagree

□Uncertain

□Somewhat agree

□Very agree

8.I can provide examples and evidence of the application of telemedicine service models in community and home settings.

□Very disagree

□Somewhat disagree

□Uncertain

□Somewhat agree

□Very agree

9.I have discussed the advantages of telehealth with other telehealth providers or patients.

□Very disagree

□Somewhat disagree

□Uncertain

□Somewhat agree

□Very agree

10.I would like to spend time thinking about how to better implement telehealth services.

□Very disagree

□Somewhat disagree

□Uncertain

□Somewhat agree

□Very agree

11.I believe that telehealth services can solve the problems caused by scheduling and excessive workload.

□Very disagree

□Somewhat disagree

□Uncertain

□Somewhat agree

□Very agree

12.I can use telehealth service equipment 24 hours a day.

□Very disagree

□Somewhat disagree

□Uncertain

□Somewhat agree

□Very agree

13.I believe that telehealth services can be included in medical insurance.

□Very disagree

□Somewhat disagree

□Uncertain

□Somewhat agree

□Very agree

14.I confirm that telehealth service equipment is reliable and has good tele-technical support and emergency plans.

□Very disagree

□Somewhat disagree

□Uncertain

□Somewhat agree

□Very agree

15.In applying telehealth services, I can access established and reliable clinical consultation networks/experts that are available.

□Very disagree

□Somewhat disagree

□Uncertain

□Somewhat agree

□Very agree

16.Through telehealth services, I can gain useful clinical knowledge and continuing medical education (CME).

□Very disagree

□Somewhat disagree

□Uncertain

□Somewhat agree

□Very agree

17.When applying for telehealth services, I must address responsibility issues and professional qualifications.

□Very disagree

□Somewhat disagree

□Uncertain

□Somewhat agree

□Very agree
