# Supplementary material for: Exploring the telehealth readiness and its related factors among palliative care specialist nurses: a cross-sectional study in China
Source: BMC Palliat Care. 2023 Jun 28;22:82. doi: 10.1186/s12904-023-01209-1 (PMC10303327; doi:10.1186/s12904-023-01209-1)
Supplement: Supplementary file 3 — Additional file 3. [file 12904_2023_1209_MOESM3_ESM.docx]

**Appendix III Chinese version of the Innovative Self-Efficacy Scale (ISES)**

1.I can accomplish the majority of my goals through innovative ways of working.

□Very disagree

□Disagree

□Neutral

□Agree

□Very agree

2.Faced with difficult tasks, I'm pretty sure I'll be able to do it creatively.

□Very disagree

□Disagree

□Neutral

□Agree

□Very agree

3.Overall, I feel that I can innovatively make significant achievements.

□Very disagree

□Disagree

□Neutral

□Agree

□Very agree

4.Most of the time, I can turn an innovative idea into a reality.

□Very disagree

□Disagree

□Neutral

□Agree

□Very agree

5.I can deal with all kinds of challenges creatively.

□Very disagree

□Disagree

□Neutral

□Agree

□Very agree

6.I am confident in my creative ability to accomplish various tasks.

□Very disagree

□Disagree

□Neutral

□Agree

□Very agree

7.Compared to others, I am very creative in my work.

□Very disagree

□Disagree

□Neutral

□Agree

□Very agree

8.Even when things are difficult, I still do it creatively.

□Very disagree

□Disagree

□Neutral

□Agree

□Very agree
